# Affective Language, Interpretation Bias and Its Molecular Genetic Variations: Exploring the Relationship Between Genetic Variations of the OXTR Gene (rs53576 and rs2268498) and the Emotional Evaluation of Words Related to the Self or the Other

**DOI:** 10.3389/fpsyg.2019.00068

**Published:** 2019-02-20

**Authors:** Friedrich Meixner, Christian Montag, Cornelia Herbert

**Affiliations:** ^1^Applied Emotion and Motivation Psychology, Institute of Psychology and Education, Ulm University, Ulm, Germany; ^2^Molecular Psychology, Institute of Psychology and Education, Ulm University, Ulm, Germany; ^3^The Clinical Hospital of Chengdu Brain Science Institute, MOE Key Lab for Neuroinformation, University of Electronic Science and Technology of China, Chengdu, China

**Keywords:** OXTR, social cognition, language, self, emotional evaluation, rs53576, interpretation bias

## Abstract

Several studies have demonstrated links between oxytocin and socio-emotional information processing. Regarding the frequently studied single nucleotide polymorphism (SNP) rs53576 and the less studied, functional polymorphism rs2268498 of the oxytocin receptor (OXTR) gene, previous research suggested that their variants might be associated with different proficiency in the processing of social information. Differences between the genotype variants are not restricted to non-verbal stimulus processing but have also been reported in the verbal domain. Moreover, there is evidence that oxytocin expression influences empathic communication and language development during childhood, indicating that language-based theory-of-mind abilities may be affected by interindividual differences in OXTR genotypes as well. The present study therefore investigates whether two prominent SNPs of the OXTR gene (rs53576 GG vs. A+; rs2268498 TT vs. C+) also play a role in the affective evaluation of verbal stimuli varying in emotional valence and in self-other reference. Participants (*N* = 149 Caucasian participants, 104 females; A+: *n* = 80, GG: *n* = 69; C+: *n* = 98, TT: *n* = 51) were presented a series of written, self-, other-, and unreferenced words of positive, negative, and neutral valence and asked to affectively evaluate each word pair as positive, negative, or neutral by button press. In line with previous research, reaction times and accuracy (number of valence-congruent responses) showed a self-positivity bias (i.e., preferential processing of self-related positive words), which, however, was unaffected by participants’ genotype. Regarding affective evaluation of neutral words (interpretation bias), A+ carriers displayed a weaker positive interpretation bias compared to GG carriers in the other– and unreferenced stimulus categories. C+ carriers showed a weaker positive interpretation bias than TT carriers in the self-reference condition and in the other-reference condition. These effects were independent from participants’ gender. The present results suggest that the OXTR genotype and hence participants’ genetic oxytocin sensitivity may cause an interpretation bias in the spontaneous appraisal of neutral words.

## Introduction

Oxytocin is a neuropeptide of the brain. In mammals, oxytocin has been shown to play a role in social bonding, attachment and the formation of interpersonal and maternal relationships. Because of its strong associations with bonding and mating behavior, it has been labeled the “love hormone” (Bakermans-Kranenburg and van IJzendoorn, 2013), which might be an oversimplification, because oxytocin is involved in several processes related to social cognition (Ebstein et al., 2010; MacDonald and MacDonald, 2010; Kumsta and Heinrichs, 2013). Regarding its influence on socioemotional behavior, oxytocin cannot only act peripherally as a hormone, but also centrally as a neuropeptide and neuromodulator. It is mainly synthesized in the central structures of the paraventricular (PVN) and the supraoptical nuclei (SON) of the hypothalamus, projecting to the pituitary gland, from where oxytocin is subsequently released into the bloodstream. Moreover, the PVN has various neural projections to brain areas involved in the processing of social and emotional information such as the amygdala and the hippocampus, i.e., limbic brain structures also possessing a rich number of oxytocin receptors (OXTRs) (Landgraf and Neumann, 2004; MacDonald and MacDonald, 2010; Bakermans-Kranenburg and van IJzendoorn, 2013).

To investigate the effects of oxytocin on human behavior, the following two experimental approaches have been employed most frequently in previous research: (a) measuring or manipulating oxytocin levels by administrating oxytocin intranasally or intravenously to determine its direct impact on human behavior and (b) measuring genetic individual differences in single nucleotide polymorphisms (SNPs) located in the genome on the chromosome 3p25 and related to the coding of the OXTR gene. Basically, SNPs are the most common form of genetic variation in humans.

Regarding intranasal administration of oxytocin, an increase in positive self-attribution in men (Colonnello and Heinrichs, 2014), increased trust (Kosfeld et al., 2005), increased attention to positive social information (Ditzen et al., 2009; Domes et al., 2013), as well as an attenuated neural reactivity in the amygdala and the visual cortex (fusiform gyrus) towards threatening face cues (Kanat et al., 2015) could be shown repeatedly following experimental oxytocin administration. This supports the role of oxytocin in socio-emotional signal and information processing.

### Oxytocin and Its Genetic Variations in the Oxytocin Receptor Gene (OXTR rs53576, rs2268498)

Regarding individual differences in the SNPs of the OXTR gene a plethora of studies in humans already reported associations with social behaviors and social information processing. In these studies, two SNPs – rs53576 and rs2268498 – emerged as very promising genetic candidates contributing to individual differences in social information processing. Although located on an intron region of the OXTR gene and therefore not expressed in the final messenger RNA, the SNP rs53576 has been investigated very frequently in previous studies. The SNP rs53576 can occur in three different allele variants called AA, AG, and GG. Regarding the distribution of these three variants in the general population, the literature shows a trend towards higher prevalence of GG carriers, particularly in European-American samples (Kim et al., 2010) in which the distribution of AA, AG, and GG carriers roughly corresponds to percentages of 12% (AA), 38% (AG), and 50% (GG), respectively. While some studies report no differences between different ethnic groups in the occurrence of GG/GA/AA-allele-carriers (e.g., Rodrigues et al., 2009) in Korean, Korean-American and European-American samples (Kim et al., 2010), other studies found the above reported frequency distributions only in European-American samples. Because of the genotype’s skewed distribution, the three different variants have been clustered into A+ (AA/AG) and GG carriers in many previous publications. However, a direct influence of these variations (A+ vs. GG) on OXTR functionality has yet to be proven in humans (for animals, see for example King et al., 2016). Nevertheless, from an evolutionary perspective, the different genetic variations in the OXTR gene might have adaptive value for human behavior as well, i.e., in some situations one variant of the genotype might be superior compared to the other genotype variant and vice versa in other situations.

In line with these speculations, previous studies which investigated the relationship between individual variations in the rs53576 genotype and behavior found that A+ carriers score lower on positive affect (male A+ carriers only; Lucht et al., 2009), show lower non-verbal intelligence (Lucht et al., 2009), less support seeking when in distress (Kim et al., 2010), lower dispositional empathy (Rodrigues et al., 2009), higher depression scores (Saphire-Bernstein et al., 2011), increased harm avoidance (females only; Wang et al., 2014), and global deficits in the processing of social information compared to the GG genotype. For example, Rodrigues et al. (2009) could demonstrate increased scores in the “Reading the Mind in the Eyes” test (RMET) in GG carriers compared to carriers of the A+ genotypes. The RMET is considered to be a reliable measure of accurate emotion recognition. Thus, GG carriers seem to be more accurate in the recognition of emotions expressed by other people. Besides differences in emotion recognition, GG carriers have also been found to show lower physiological and dispositional stress reactivity compared to A+ carriers (Rodrigues et al., 2009) and to be more sensitive towards social exclusion (McQuaid et al., 2015). Again, this implies that GG carriers might be more sensitive in the processing of socio-emotional information compared to A+ carriers. In accord with these findings, other studies (e.g., Smith et al., 2014) reported higher levels of empathic concern and sympathetic arousal, as measured by increased electrodermal activity, in GG carriers as opposed to A+ carriers during the perception of harm dealt to another person. For more complex effects of OXTR rs53576 on empathy please see a recent molecular genetic association study with data from China and Germany (Montag et al., 2017). As with studies employing intranasal administration of oxytocin, some molecular genetic studies dealing with the OXTR gene also report gender-based differences (e.g., Lucht et al., 2009; Wang et al., 2014; Feng et al., 2015). For example, Feng et al. (2015) investigated individual variations in OXTR rs53576 together with intranasal application of oxytocin in a functional imaging study while participants performed a reciprocal cooperation game. The authors report increased activation of the left ventral caudate nucleus in male GG carriers after intranasal oxytocin treatment compared to decreased activation in female carriers, possibly suggesting an inverted U-shape curve of the dose–response relationship between central oxytocin, which is higher in females at baseline levels, and the neural activation in reward-related structures such as the left ventral caudate nucleus following reciprocated cooperation.

Although rs53576 might be one of the most studied OXTR polymorphisms with respect to differences in socio-emotional information processing, recent research also highlights a role of the OXTR rs2268498 polymorphism. In contrast to the rs53576, the SNP rs2268498 is located in the adjacent area of the OXTR gene’s promoter region. Genetic variations of both the promoter and exon regions of a gene can exert direct influence on a functional level and by this alter the gene product in terms of different enzyme activity (as observed for the prominent COMT Val158Met polymorphism; see review by Montag et al., 2012) or on receptor density (as has been demonstrated for the DRD2/ANKK1 Taq Ia polymorphism; for a discussion see Comings, 1999). In line with this, in humans genetic variations in the SNP rs2268498 have already proven to be functional (Reuter et al., 2017). The SNP rs2268498 also occurs in three different genotype variations: CC, CT and TT, respectively, and akin to the SNP rs53576, its distribution in the population varies (Caucasian samples showing 20.1% CC, 52.1% CT, and 27.7% TT; Montag et al., 2017). Although the genotype status of the SNPs rs53576 and rs2268498 can covary (e.g., being A+ carrier might also be associated with being C+; Laursen et al., 2014), previous studies investigating variations in both SNPs suggest that their relationship with social behavior may not be mutually interchangeable. Individual variations in rs2268498 have been associated with autism spectrum disorder (Montag et al., 2017), differences in interpersonal perception (Melchers et al., 2015), differences in emotion recognition skills (Melchers et al., 2013) and other social abilities related to perspective taking, such as moral judgment (Walter et al., 2012). Moreover, recent genetic imaging studies found differences in C+ vs. TT carriers in resting state connectivity between amygdala subregions and fusiformis/inferior occipital cortex (Zimmermann et al., 2018), supporting the functional relationship between variations in this SNP in neural pathways critical for automatic processing of socio-emotional information including the processing of verbal emotional content (e.g., Herbert et al., 2009).

### Influence of OXTR Polymorphisms on Verbal Affective Information Processing

While many previous genetic studies investigated non-verbal social and affective processing, the above illustrated relations between oxytocin and social behavior on the one hand and oxytocin-related genotype differences and social behavior on the other hand may not be restricted to differences in non-verbal social and affective processing, but could also hold true for linguistic and auditory processing of social and emotional information: for example, in a very recent study (Pfundmair et al., 2016), it could be shown that oxytocin (intranasal application) facilitates the comprehension of affective verbal communication. Regarding OXTR polymorphisms, Tops et al. (2011) analyzed differences between A+ and GG carriers in self-reported difficulties in hearing and understanding others in noisy environments. In line with the previous literature, GG carriers displayed greater efficiency in the processing of interpersonal information compared to carriers of the A+ genotype. Thus, the latter two studies suggest a link between oxytocin or individual variation in OXTR genotypes and social information processing during verbal communication. However, the results of these studies are based solely on questionnaire data, underlining the necessity to employ experimental paradigms that consist of linguistic stimuli to better understand how the processing of social and emotional information particularly related to semantics and language could be influenced by OXTR polymorphisms.

Regarding the interplay between OXTR genotypes and language processing, research on autism spectrum disorder has accumulated some evidence that autism-related difficulties in interpersonal information processing and in theory-of-mind (ToM) abilities – especially in those ToM abilities requiring language comprehension and higher-order cognitive and semantic understanding – can be positively influenced by intranasal/intravenous oxytocin applications (Hollander et al., 2007; Dadds et al., 2014). It has been hypothesized that the amygdala, an OXTR-dense area of the brain, plays an important role in the processing of emotional and social information and in the development of autism spectrum disorders (Baron-Cohen et al., 2000; Janak and Tye, 2015; Herrington et al., 2016). Moreover, regarding language processing, there is increasing evidence from emotional word processing studies (for an overview, see Herbert et al., 2018a) that in the brain and the body, language is closely linked to emotions and emotional word processing is associated with changes in neural activation of emotional brain structures: for instance, during reading, words of emotional content and of high self-relevance have been found to elicit changes in neural activity in the amygdala, the insula and related emotional brain structures involved in the recognition and evaluation of affective and personally relevant information (e.g., ventromedial prefrontal cortex; Herbert et al., 2009, 2011b).

### Verbal Affective Information Processing: Self-Positivity Bias and Positive/Negative Interpretation Bias

In healthy individuals, the processing of neutral and emotional verbal stimuli is indeed already well-documented in a wealth of studies (e.g., see Herbert et al., 2018a, for an overview): in particular, it has been shown that when exposed to verbal stimuli, healthy participants display a bias for self-referenced information (e.g., Esslen et al., 2008; Zhou et al., 2010; Blume and Herbert, 2014) and often also a positivity bias (e.g., Herbert et al., 2008, 2009; Augustine et al., 2011). These observations are well in line with the well-known self-reference effect (Symons and Johnson, 1997) and the observation of self-serving attributional biases in affective processing (Mezulis et al., 2004). Interestingly, when words possess both emotional content and self-reference, processing of self-related emotional words reveals a self-positivity bias on a behavioral and a neural processing level (Fossati et al., 2003; Herbert et al., 2011a; Fields and Kuperberg, 2016; Weis and Herbert, 2017; Meixner and Herbert, 2018). This suggests that the combination between positive emotional valence and self-reference significantly increases the personal relevance of the meaning of a stimulus and consequently enhances the reaction towards it. In other words, when healthy participants are presented a series of emotional and neutral words that are related either to their own self or the self of another person and asked to judge these words in terms of their emotional valence and concomitant feelings, they judge positive words more often as positive and thus as valence-congruent when related to the self as compared to when related to another person. Moreover, they are faster in this decision than in their decisions to self-related neutral or negative words (e.g., Weis and Herbert, 2017; Meixner and Herbert, 2018). Although quite robust, this self-positivity bias can be subject to inter- and intraindividual variations: it can be significantly extended from the first person to the third person in individuals experiencing a romantic relationship (e.g., Meixner and Herbert, 2018; Quintard et al., 2018), whereas in participants with mental disorders, this self-positivity bias may be significantly attenuated, absent or even changed towards a negativity bias (Herbert et al., 2014, 2018b; Winter et al., 2015, 2018). So far, however, one can only speculate how variations in OXTR genotypes might influence these interpretation biases.

Yet a further example for biases in affective evaluation and interpersonal communication is the so-called *interpretation bias*, i.e., the tendency to interpret semantically ambiguous and neutral information in a positive or negative way. So far, the interpretation bias has been studied most frequently in association with depressive symptomatology (Wisco and Nolen-Hoeksema, 2010; Berna et al., 2011) and social anxiety (Amin et al., 1998; Huppert et al., 2003; Amir et al., 2005), showing a strong tendency towards negatively biased evaluation in healthy individuals as well as in patients scoring high on anxiety and depressive disorder. Regarding depressive symptoms, Wisco and Nolen-Hoeksema (2010) found evidence for a negative interpretation bias that is dependent on the specific type of “other” (i.e., an unknown vs. a familiar other person). Since close others might be more integrated in one’s self-concept and considering previous findings on behavioral differences related to OXTR genotypes outlined under the sections “Oxytocin and Its Genetic Variations in the Oxytocin Receptor Gene (OXTR rs53576, rs2268498)” and “Influence of OXTR Polymorphisms on Verbal Affective Information Processing,” an interpretation bias (positive or negative) could also be present in healthy individuals varying in OXTR genotypes when evaluating neutral stimuli differing in self-other reference.

Theoretically, processing of interpersonal information is a dynamic process that comprises different stages of information processing. According to the Component Process Model of Emotions (CPM) by Scherer (2001), several appraisal steps are completed in a sequential fashion while encountering stimuli varying in emotional content and in personal relevance. At first, stimuli are appraised for their novelty and their personal relevance for the own person including an intrinsic pleasantness check of the stimulus’ content in terms of its unpleasantness or pleasantness. Secondly, the implications or consequences a stimulus or an event has for the person are assessed. In a third step, the own person’s coping potential is considered, which is then followed by an evaluation of its normative significance with respect to one’s own self-concept or to certain social norms. Difficulties in interpersonal processes and in the processing of socio-emotional information (be it non-verbally or verbally conveyed), as have been demonstrated for certain OXTR genotypes (see the sections “Oxytocin and Its Genetic Variations in the Oxytocin Receptor Gene (OXTR rs53576, rs2268498)” and “Influence of OXTR Polymorphisms on Verbal Affective Information Processing”), might be caused by unfavorable appraisal in any of those steps, leading to a cascade of false interpretations, which are based on wrong assumptions. This might further lead up to unjustified behavior that might provoke harsh reactions from surrounding others. However, to properly assess the mechanism of these genotype-related deficits, separate evaluation of any step of stimulus appraisal is crucial.

### Aim and Hypotheses

The aim of this study was to investigate the interplay between verbally conveyed social information processing and OXTR genotypes. In particular, and extending previous research, we thought to elucidate the role of individual differences in the OXTR genotypes (rs53576 – GG vs. A+; rs2268498 – C+ vs. TT) in affective evaluation of verbal stimuli varying in emotional valence and in self-other reference. Regarding the mechanism underlying the interplay between genes, affect and language, we were particularly interested in whether affective stimulus appraisal – in particular the appraisal of self-relevance including the intrinsic pleasantness check as proposed by the CPM (Scherer, 2001) – would be significantly affected by individual differences in the OXTR genotype. Recent studies investigating the time course of stimulus appraisal for words varying in emotional valence and in self-reference suggest that for linguistic stimuli an interaction between stimulus valence and stimulus reference occurs at later stages of information processing that are associated with in-depth elaboration of the stimulus meaning. This supports the notion that appraisal of self-relevance occurs temporally after each information (appraisal of the emotionality of the word and appraisal of its self-reference) has been checked and integrated (Herbert et al., 2011a,c; Fields and Kuperberg, 2016). Accordingly, on a behavioral level (e.g., Weis and Herbert, 2017; Meixner and Herbert, 2018), affective evaluation of words varying in both emotional content and in self-reference should elicit considerably longer reaction times necessary for the sequence of elaborations associated with the appraisal of the word’s personal relevance. Differences in the appraisal of the word’s personal relevance related to individual differences in the OXTR genotypes (rs53576 – GG vs. A+, rs2268498 – C+ vs. TT) may manifest in differences in response accuracy as well as in differences in reaction times.

Moreover, since previous genetic studies focused mainly on the effects of oxytocin on the processing of positive and/or negative stimuli, the present study also examined whether affective evaluation of neutral stimuli varying in self-other reference will differ as a function of individual differences in OXTR genotypes. Neutral stimuli are devoid of any emotional quality. Thus, when appraised according to their personal relevance and when presented with or without self-other reference, their content may become susceptible to an *interpretation bias*, facilitating valence-incongruent judgments, e.g., evaluating neutral stimuli either as positive or as negative.

The relationship between individual differences in OXTR genotypes and affective evaluation of verbal stimuli varying in emotional and neutral content and in self–other reference has not been investigated before. Therefore, the following hypotheses can be based only on the results of previous genetic studies outlined in detail above. In accord with these studies, it could be expected that individual differences in both OXTR genotypes (rs53576 and rs2268498) might contribute to differences in affective evaluation of self- vs. other-related emotional and neutral verbal stimuli. Given that A+ carriers have been shown to exhibit greater difficulties in the categorization of other-related stimulus categories compared to GG carriers, A+ carriers should show significantly less response accuracy (i.e., valence-congruent answers) and increased reaction times in the affective evaluation of other-related words compared to GG carriers. Moreover, it could be hypothesized that A+ carriers would also tend to evaluate semantic stimuli of neutral content differently from GG carriers, probably exhibiting an *interpretation bias*, evident in valence-incongruent judgments to neutral words varying in self-other reference. Regarding individual variations in rs2268498, behavioral results are still scarce. However, due to the possible functional role of this SNP, it seems reasonable to test the same hypotheses for carriers of C+ vs. TT as well.

## Materials and Methods

### Participants

#### Recruitment and Genotype Distribution

Participants were recruited via mailing lists and advertisements around the city of Ulm and the campus of Ulm University, Germany, where all measurements took place. In line with previous OXTR genetic studies, inclusion criteria for participation were carefully chosen to exclude possible confounding factors. Therefore, participants of the present study could take part in the study only if they were Caucasian, heterosexual, 18–30 years of age, neither married nor engaged ever in their lifetime and if they had no children. In addition, to be included in the current data analysis, participants had to be either already registered in the Ulmer genetic database led by the Department of Psychology and Education, Molecular Psychology at the University of Ulm, Germany or become a voluntary member thereof. For the present study, *N* = 175 volunteers, all members or willing to register in the genetic database could be included in the present data analysis. The *N* = 175 participants could be taken from a larger sample of *N* = 359 participants, who over the period of 2 years all took part in behavioral experiments employing the His-Mine paradigm and the same set of stimuli and the same set of questionnaires and exclusion and inclusion criteria. Participants included in this larger project including the participants of the current data analysis all gave informed consent and received course credit or 10 euros in return for their participation. Consent obtained from all participants was both written and informed and the project and individual study and experimental design were approved by the local ethics committee of Ulm University.^1^

One participant had to be excluded from the present sample due to having reported suffering from attention deficit syndrome; two participants had to be excluded due to having reported treatment for acute psychiatric disorders. Although not reporting diagnosis of clinical depression, *n* = 6 participants had to be excluded due to self-reported depression on the Beck-Depression Inventory indicating medium-to-severe depressive symptomatology (BDI scores > 20). In addition, *n* = 17 participants had to be excluded since they reported being non-native speakers of German and/or being left-handed or ambidextrous.^2^

The remaining sample of participants included in the analysis comprised *N* = 149 healthy adults (104 females, 44 males, 1 unspecified), with a mean age of *M* = 22.19 years, *SD* = 2.52, all right-handed and native speakers of German.

Genotype distribution in the final sample was in Hardy–Weinberg equilibrium for both SNPs (rs53576: χ^2^ = 0.20, *p* = 0.65; rs2268498: χ^2^ = 1.47, *p* = 0.22), corresponding to AA: *n* = 17, AG: *n* = 63, GG: *n* = 69, and CC: *n* = 32 CT: *n* = 66 or TT: *n* = 51) and was in line with reports from previous studies (e.g., Walter et al., 2012; Melchers et al., 2013; Montag et al., 2017). Also, in line with previous studies was the conjunct distribution of both SNPs [A+/C+: *n* = 72; A+/TT: *n* = 8; GG/C+: *n* = 26; GG/TT: *n* = 43; χ^2^(1) = 45.05, *p* < 0.001]. Because of the skewed distributions and in accord with previous research (e.g., Rodrigues et al., 2009; Saphire-Bernstein et al., 2011; Tops et al., 2011; Feng et al., 2015), AA/AG-genotypes as well as CC/CT-genotypes were combined into one group of A+ carriers or C+ carriers, resulting in comparable group sizes of *n* = 80 (A+) and *n* = 69 (GG) participants for the SNP rs53576 and *n* = 98 (C+) and *n* = 51 (TT) participants for the SNP rs2268498. In the following analyses, A+ genotypes will be compared with the GG genotype and the C+ genotypes will be compared with the TT genotype. The A+ and GG-genotype distribution as well as the C+ and TT genotype distribution did not differ by gender, [A+/GG: χ^2^(1) = 0.006, *p* = 0.938; C+/TT: χ^2^(1) = 0.108, *p* = 0.742].

### Genetic Sampling

All genetic sampling was done non-invasively via buccal swaps. DNA extraction was performed on MagNA Pure 96 using a commercial extraction kit (Roche, Mannheim, Germany). Genotyping was performed on a Cobas Z 480 Light Cycler via detection of melting curves. The simple probes were designed by TIB MolBiol (Berlin, Germany).

### Materials

**FIGURE 1:**
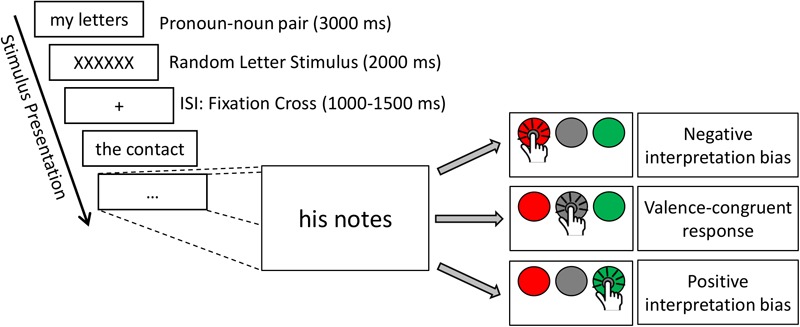
Time course of the experimental paradigm with different response types, illustrating positive, and negative interpretation biases.

In the present study, a modified version of the affective His-Mine paradigm was used (Herbert et al., 2011a,c, 2018b; Blume and Herbert, 2014; Winter et al., 2015, 2018; Weis and Herbert, 2017). The affective His-Mine paradigm consists of emotional and neutral nouns that are paired with possessive pronouns of the first or third person as well as with articles (Herbert et al., 2018b; Meixner and Herbert, 2018) or negated pronouns (Weis and Herbert, 2017) devoid of any personal reference. As shown in Figure 1, in the present study the experimental design consisted of three different self-reference and emotion conditions: self-related and other-related^3^ emotional and neutral pronoun-noun pairs (e.g., my fear, my happiness, my office) and emotional and neutral article-noun pairs devoid of self- or other-reference. In total (see also Meixner and Herbert, 2018), 60 nouns were included with 20 nouns per emotional valence category (positive, negative, and neutral). These 60 nouns were the same for each reference category (self-, other-, and no reference) to reduce stimulus variance across stimulus categories. Nouns were matched according to normative ratings of valence and arousal. Nouns and ratings were taken from our own word corpus (e.g., Herbert et al., 2006, 2008; Kissler et al., 2006, 2007) providing for 469 German adjectives and 311 German nouns emotional valence and emotional arousal ratings on the 9-point Self-Assessment Manikin (SAM; Bradley and Lang, 1994) and for concreteness on 9-point Likert scales. Affective ratings of this word corpus are also provided in other standardized German datasets of affective words, using slightly different procedures and rating scales (e.g., see Berlin Affective Word List, BAWL-R, Võ et al., 2009). In addition, nouns were also matched for linguistic dimensions, e.g., word length and word frequency to avoid confounding effects with linguistic dimensions. Thus, the final sample of word material differed only in valence and arousal, i.e., positive and negative words eliciting higher arousal than neutral nouns, and positive, negative, and neutral nouns differing significantly in valence from each other, as can be seen in Table 1.^4^

**Table 1:** Descriptive statistics of the different word categories used in this study.

	Valence	Arousal	Concreteness	Length	Frequency
Positive	7.31 (*0.72*)^a^	4.68 (*0.74*)^a^	4.68 (*0.70*)^a^	5.75 (*1.71*)^a^	263.15 (*283.53*)^a^
Neutral	5.34 (*0.67*)^b^	2.43 (*0.97*)^b^	4.05 (*1.83*)^a^	6.35 (*1.35*)^a^	227.00 (*253.77*)^a^
Negative	2.56 (*0.53*)^c^	4.77 (*0.69*)^a^	4.33 (*0.89*)^a^	6.30 (*1.69*)^a^	206.80 (*189.50*)^a^

*Table is taken from Meixner and Herbert (2018). Different superscripted letters a, b, and c indicate statistically significant differences between word categories regarding the respective dimensions (*p* ≤ 0.05); mean values are depicted, values in brackets represent standard deviations. Word length is expressed in total characters; word frequency is expressed as appearance per one million words, for further information, please see the section “Materials”.*

Each stimulus pair (pronoun–noun or article–noun pair) was presented in one trial, resulting in a total of 180 trials. Each trial started with a fixation cross displayed for a random interval between 1000 and 1500 ms. Word stimuli were presented on a computer screen with a resolution of 1280 × 1024 pixels at a refresh rate of 60 Hz, in black 70 pt letters (Times New Roman) on a white background. Participants were seated at a comfortable viewing distance of 60 cm, resulting in a visual angle of 1° 54° 0.58° (stimulus height). Stimuli remained on the screen for 3000 ms or until participants pressed any of the answer keys. After each verbal stimulus, a visual stimulus consisting of six letters (XXXXXX) was presented for 2000 ms to diminish any carry over effects from one trial to the next. Presentation order of the stimuli was randomized for each participant to avoid sequence effects.

### Procedure

Upon arrival, participants read and signed the consent form and answered basic demographic questions about their age, gender, mother language including specific anamnestic variables asking for visual/acoustic impairments, history of neurological/ psychiatric disorders, alcohol/drug abuse, etc. Subsequently, participants received detailed written and oral instructions from the experimenter regarding the experimental paradigm. They were told that they will be presented a series of words that could describe emotions or objects belonging either to themselves, to a third person or that contained no personal reference (article–noun conditions). Participants were instructed to evaluate those stimuli using one of three keyboard buttons. Keyboard buttons were color-coded in green (positive) or red (negative) or uncolored (neutral). Key presses had to be given with the index finger of the dominant hand, resting on a fixed starting position, equidistant to the three target buttons. Furthermore, participants were instructed to respond spontaneously based on their feelings elicited during reading and – based on these – as fast and as accurate as possible. The experimental paradigm lasted about 20 min and was programmed using Presentation^®^ software^5^ (Version 0.60, Neurobehavioral Systems, Inc., Berkeley, CA, United States).

Afterward, participants answered all self-report measures. These included measurements of depressive symptomatology (Beck-Depression Inventory, BDI-II, German version by Hautzinger et al., 2009), anxiety (State-Trait-Anxiety Inventory, STAI, Laux et al., 1981), different facets of self-concept (Deusinger, 1986), and empathy (Paulus, 2009; based on the Interpersonal Reactivity Index, IRI by Davis and Oathout, 1992). Furthermore, we included a measure of alexithymia (Toronto Alexithymia Scale, TAS-20, Bagby et al., 1994; German translation by Bach et al., 1996), since the inability to describe or identify one’s own emotions might be related to the evaluation of self-related stimuli. In total, the complete experiment including the His-Mine paradigm, self-report questionnaires and genetic sampling (buccal swaps) lasted about 60 min.

A detailed overview on the descriptive data including participants’ self-report data (age, gender, depression, anxiety, empathy, and alexithymia scores) as a function of their genotype is provided in Table 2.

**Table 2:** Demographic data and BDI-II, STAI, empathy, and alexithymia scores (all calculated as sum scores as suggested by the respective manuals) of the sample, M (SD), including OXTR rs53576 A+ carriers (first line) and GG carriers (second line), and OXTR rs2268498 C+ carriers (third line) and TT carriers (fourth line).

		Gender (number of
	Age (years)	female participants)	BDI-II	STAI (*state*)	STAI (*trait*)	Empathy	Alexithymia
A+ (*n* = 80)	21.99 (*2.21*)*^a^*	*n* = 56*^a^*	5.67 (*6.55*)*^a^*	35.19 (*7.03*)*^a^*	39.03 (*8.40*)*^a^*	53.16 (*7.62*)*^a^*	46.19 (*9.80*)*^a^*
GG (*n* = 69)	22.42 (*2.84*)*^a^*	*n* = 48*^a^*	5.25 (*5.06*)*^a^*	35.39 (*7.88*)*^a^*	38.16 (*9.59*)*^a^*	53.51 (*6.50*)*^a^*	45.47 (*10.69*)*^a^*
C+ (*n* = 98)	22.20 (*2.49*)*^a^*	*n* = 68*^a^*	5.66 (*4.64*)*^a^*	35.17 (*6.96*)*^a^*	38.94 (*8.66*)*^a^*	53.31 (*7.30*)*^a^*	45.92 (*9.24*)*^a^*
TT (*n* = 51)	22.16 (*2.60*)*^a^*	*n* = 36*^a^*	5.08 (*5.06*)*^a^*	35.50 (*8.27*)*^a^*	38.02 (*9.52*)*^a^*	53.35 (*6.76*)*^a^*	45.72 (*11.94*)*^a^*

*Different superscripted letters a and b would indicate statistically significant differences between groups regarding the respective dimensions (independent samples *t*-tests; gender: chi-square test of independence; *p* ≤ 0.05); sum scores are depicted, values in brackets represent standard deviations.*

### Analysis: OXTR Genotypes (rs53576 and rs2268498) and Response Accuracy (Valence-Congruent Responses) and Reaction Times

The influence of participants’ OXTR genotypes on response accuracy (i.e., number of valence-congruent responses^6^) and reaction times were statistically analyzed with repeated measures analysis of variance (ANOVA) using IBM SPSS Statistics 24 software and a 3 × 3 × 2 full factorial design for each SNP separately. The full factorial design included the factors *stimulus valence* (positive, negative, neutral), *stimulus reference* (self, other, no personal reference) as within-subject factors and *genotype* (A+/GG or C+/TT) as between-subject factor. Reaction times were kept in their raw units (ms) and, unless otherwise specified (see interpretation bias), only valence-congruent trials were included in the calculation of mean reaction times.

If participants didn’t respond to the word categories in a valence-congruent way (e.g., evaluating neutral stimuli such as “my furniture” with a neutral button press), mean reaction times for this category could not be computed. Consequently, these participants were excluded from ANOVAs using reaction times as the dependent variable, which also results in different degrees of freedom compared to ANOVAs analyzing response accuracy, where 0 is a valid score.

Degrees of freedom were corrected using the Greenhouse–Geisser correction, if sphericity was violated. Significant within-subject effects in the ANOVA designs were further analyzed in *post hoc* tests using paired samples *t*-tests, significant between-subject effects were analyzed using independent samples *t*-tests. To assess a possible self-positivity bias and potential interactions with OXTR *genotype, post hoc* tests of reaction times and response accuracy have been calculated, comparing positive words with self-reference to positive words with other-reference and to negative/neutral words with self-reference (e.g., Meixner and Herbert, 2018). *Post hoc* tests of OXTR genotype-related differences in affective evaluation of other-related words consisted of independent samples *t*-tests of reaction times and response accuracy comparing A+ to GG carriers (rs53576; one-sided testing), and C+ to TT carriers (rs2268498; two-sided testing), respectively. If homogeneity of variances could not be assumed, Welch’s *t*-test is reported instead. Measures of effect size (partial eta square, $\eta_{p}^{2}$) are reported for the ANOVAs regarding OXTR genotypes.

### OXTR Genotypes (rs53576 and rs2268498) and Interpretation Bias

For assessment of the interpretation bias, valence-incongruent responses (*positive* and *negative*) in the neutral stimulus category, regardless of stimulus reference, were summed up to check whether the total amount of valence-incongruent responses to neutral words differs as a function of participants’ genotype. Next, percentage scores were computed to determine the degree of the negative vs. positive *interpretation bias*, which was defined as the *number of times a “neutral” stimulus had been evaluated as “negative”* relative to the *total interpretation bias (neutral evaluated as negative + neutral evaluated as positive)*. A high score (>0.5) therefore indicates a negativity bias in the reaction towards neutral words, whereas a low score (<0.5) indicates a positivity bias.

Analysis of variance and independent samples *t*-tests (one-sided testing) were performed to further examine the relation between the rs53576 *genotype* (A+ vs. GG) or the rs2268498 genotype (C+ vs. TT), and the positive or negative tendency of the *interpretation bias* (as described above).

### OXTR rs53576, rs2268498 and Self-Report Measures

To determine whether OXTR genotypes show relations with self-reported empathy, empathic concern, mood states including depressive symptoms and anxiety, independent samples *t*-tests (see also Table 2) and correlation analyses were performed (Pearson correlation, two-sided testing with *p* ≤ 0.05 as significance criteria).

## Results

### Overall Response Accuracy, Reaction Times, and OXTR Genotypes

Since every stimulus category consisted of 20 pronoun–noun pairs, a maximum number of 20 valence-congruent responses was possible in each of the 3 (*stimulus valence*) × 3 (*stimulus reference*) categories. Mean accuracy of the sample was *M* = 15.53 (*SD* = 2.61), which equals 77.65% response accuracy across all categories, indicating accuracy well above guessing levels (33.3%). Mean reaction time in milliseconds was *M* = 1248.91 ms (*SD* = 293.65) across all categories. Overall reaction time data and response accuracy for each stimulus category are listed in Table 3.

**Table 3:** Reaction times in milliseconds (upper part) and response accuracy (lower part) of all participants for stimuli with self-reference, other-reference, no personal reference and of positive, negative, neutral valence, M (SD).

M (*SD*)	Self-reference	Other-reference	No personal reference
**Reaction time in milliseconds**		
Positive	1131.54 (*289.20*)	1230.91 (*337.6*)	1113.54 (*257.25*)
Negative	1252.59 (*345.21*)	1206.18 (*329.38*)	1185.90 (*370.22*)
Neutral	1415.96 (*405.63*)	1455.38 (*397.24*)	1254.89 (*337.24*)
**Response accuracy (valence-congruent answers)**		
Positive	17.72 (*3.34*)	15.79 (*4.96*)	16.77 (*3.64*)
Negative	17.84 (*3.76*)	17.52 (*3.91*)	17.62 (*3.84*)
Neutral	10.30 (*4.87*)	12.03 (*5.41*)	14.19 (*4.22*)

Neither overall response accuracy nor overall reaction times differed significantly between A+ and GG carriers, or between C+ and TT carriers [rs53576: response accuracy: A+ (*M* = 15.26, *SD* = 3.03) vs. GG (*M* = 15.85, *SD* = 1.99); *t*(138) = 1.41; *p* = 0.160; reaction times in milliseconds: A+ (*M* = 1255.91, *SD* = 331.74) vs. GG (*M* = 1241.30, *SD* = 248.25); *t*(138) = 0.293; *p* = 0.770; rs2268498: response accuracy: C+ (*M* = 15.32, *SD* = 3.00) vs. TT (*M* = 15.92, *SD* = 1.58); *t*(147) = 1.59; *p* = 0.113; reaction times in milliseconds: C+ (*M* = 1269.15, *SD* = 300.28) vs. TT (*M* = 1212.49, *SD* = 280.59); *t*(138) = 1.09; *p* = 0.275].

### Self-Positivity Bias and OXTR Genotypes

#### Reaction Times and OXTR Genotype (rs53576)

The 3 (*stimulus valence*) × 3 (*stimulus reference*) × 2 (*genotype A+/GG*) repeated measures ANOVA (DV: *reaction times* in milliseconds) revealed significant main effects of the factors *stimulus valence* [*F*(1.82,252) = 71.86, *p* ≤ 0.001, $\eta_{p}^{2}$ = 0.342], and *stimulus reference* [*F*(2,276) = 71.42, *p* ≤ 0.001, $\eta_{p}^{2}$ = 0.341], as well as a significant interaction between *stimulus valence* and *stimulus reference* [*F*(3.45,476) = 22.18; *p* ≤ 0.001, $\eta_{p}^{2}$ = 0.138]. However, there were neither interaction effects between *genotype* and *stimulus valence* [*F*(1.82,251) = 1.38; *p* = 0.254, $\eta_{p}^{2}$ = 0.010] or *stimulus reference* [*F*(1.99,274) = 1.62; *p* = 0.200, $\eta_{p}^{2}$ = 0.012], nor a three-way interaction [*F*(3.45,476) = 0.195; *p* = 0.921, $\eta_{p}^{2}$ = 0.001], nor could a main effect of *genotype* be found [*F*(1,138) = 0.086; *p* = 0.770, $\eta_{p}^{2}$ = 0.001]. *Post hoc* tests revealed a self-positivity bias showing faster reaction times for self-related positive as compared to other-related positive words (self-positive (*M* = 1127.91, *SD* = 288.94) vs. other-positive (*M* = 1230.91, *SD* = 337.59); *t*(143) = 6.28; *p* ≤ 0.001), which, however, did not differ significantly between A+ and GG carriers, as is evident in the lack of an interaction effect. Notably, in contrast to expectations, reaction times of valence-congruent responses to other-related words did also not differ between A+ and GG carriers [A+ (*M* = 1302.15, *SD* = 337.67) vs. GG (*M* = 1299.13, *SD* = 280.60); *t*(138) = 0.057; *p* = 0.477].

#### Response Accuracy and OXTR Genotype (rs53576)

The 3 (*stimulus valence*) × 3 (*stimulus reference*) × 2 (*genotype A+/GG*) repeated measures ANOVA (DV: *response accuracy*) revealed significant main effects of the factors *stimulus valence* [*F*(1.51,223) = 115, *p* ≤ 0.001, $\eta_{p}^{2}$ = 0.438], and *stimulus reference* [*F*(1.75,257) = 32.88, *p* ≤ 0.001, $\eta_{p}^{2}$ = 0.183], as well as a significant interaction between *stimulus valence* and *stimulus reference* [*F*(2.74,403) = 41.11; *p* ≤ 0.001, $\eta_{p}^{2}$ = 0.219]. There were neither interaction effects between *stimulus valence* and *genotype* [*F*(1.51,223) = 0.039; *p* = 0.926, $\eta_{p}^{2}$ = 0.000], nor between *stimulus reference* and *genotype* [*F*(1.75,257) = 1.04; *p* = 0.347, $\eta_{p}^{2}$ = 0.007], nor could a significant three-way interaction [*F*(2.74,403) = 0.054; *p* = 0.978, $\eta_{p}^{2}$ = 0.000], nor could a main effect of *genotype* be found [*F*(1,147) = 1.88; *p* = 0.172, $\eta_{p}^{2}$ = 0.013]. *Post hoc* tests revealed a self-positivity bias, showing higher response accuracy for self-related positive as compared to other-related positive words [self-positive (*M* = 17.72, *SD* = 3.34) vs. other-positive (*M* = 15.79, *SD* = 4.96); *t*(148) = 5.94; *p* ≤ 0.001]. However, as is evident in the lack of an interaction effect, this did not differ significantly between A+ and GG carriers. In addition, response accuracy in other-related words did not differ significantly between A+ and GG carriers [A+ (*M* = 14.73, *SD* = 3.51) vs. GG (*M* = 15.56, *SD* = 2.45); *t*(147) = 1.63; *p* = 0.053].

#### Reaction Times and OXTR Genotype (rs2268498)

The 3 (*stimulus valence*) × 3 (*stimulus reference*) × 2 (*genotype C+/TT*) repeated measures ANOVA (DV: *reaction times* in milliseconds) revealed significant main effects of the factors *stimulus valence* [*F*(1.81,250) = 62.14, *p* ≤ 0.001, $\eta_{p}^{2}$ = 0.310], and *stimulus reference* [*F*(2,276) = 71.29, *p* ≤ 0.001, $\eta_{p}^{2}$ = 0.341], as well as a significant interactions between *stimulus valence* and *stimulus reference* [*F*(3.44,475) = 19.64; *p* ≤ 0.001, $\eta_{p}^{2}$ = 0.125]. There were no significant interaction effects between *stimulus valence* or *stimulus reference* and *genotype*, nor a three-way interaction, nor could a main effect of *genotype* be found [*F*(1,138) = 1.20; *p* = 0.275, $\eta_{p}^{2}$ = 0.009]. Comparable to the rs53576, *post hoc* tests revealed no significant differences in reaction times to self-related positive words or other-related words as a function of genotype [C+ (*M* = 1310.69, *SD* = 306.16) vs. TT (*M* = 1282.73, *SD* = 320.69); *t*(138) = 0.509; *p* = 0.612].

#### Response Accuracy and OXTR Genotype (rs2268498)

The 3 (*stimulus valence*) × 3 (*stimulus reference*) × 2 (*genotype C+/TT*) repeated measures ANOVA (DV: *response accuracy*) revealed significant main effects of the factors *stimulus valence* [*F*(1.51,222) = 99.79, *p* ≤ 0.001, $\eta_{p}^{2}$ = 0.404], and *stimulus reference* [*F*(1.75,257) = 31.64, *p* ≤ 0.001, $\eta_{p}^{2}$ = 0.177], as well as a significant interaction between *stimulus valence* and *stimulus reference* [*F*(2.75,404) = 38.84; *p* ≤ 0.001, $\eta_{p}^{2}$ = 0.209]. There were no interaction effects between *stimulus valence* and *genotype*, nor between *stimulus reference* and *genotype*, nor a three-way interaction, and no main effect of *genotype* could be found [*F*(1,147) = 1.77; *p* = 0.185, $\eta_{p}^{2}$ = 0.012]. *Post hoc* test revealed no significant differences in response accuracy to self-related positive words or other-related words as a function of individual genotype variations [C+ (*M* = 14.95, *SD* = 3.43) vs. TT (*M* = 15.42, *SD* = 2.28); *t*(138) = 0.887; *p* = 0.377].

**FIGURE 2:**
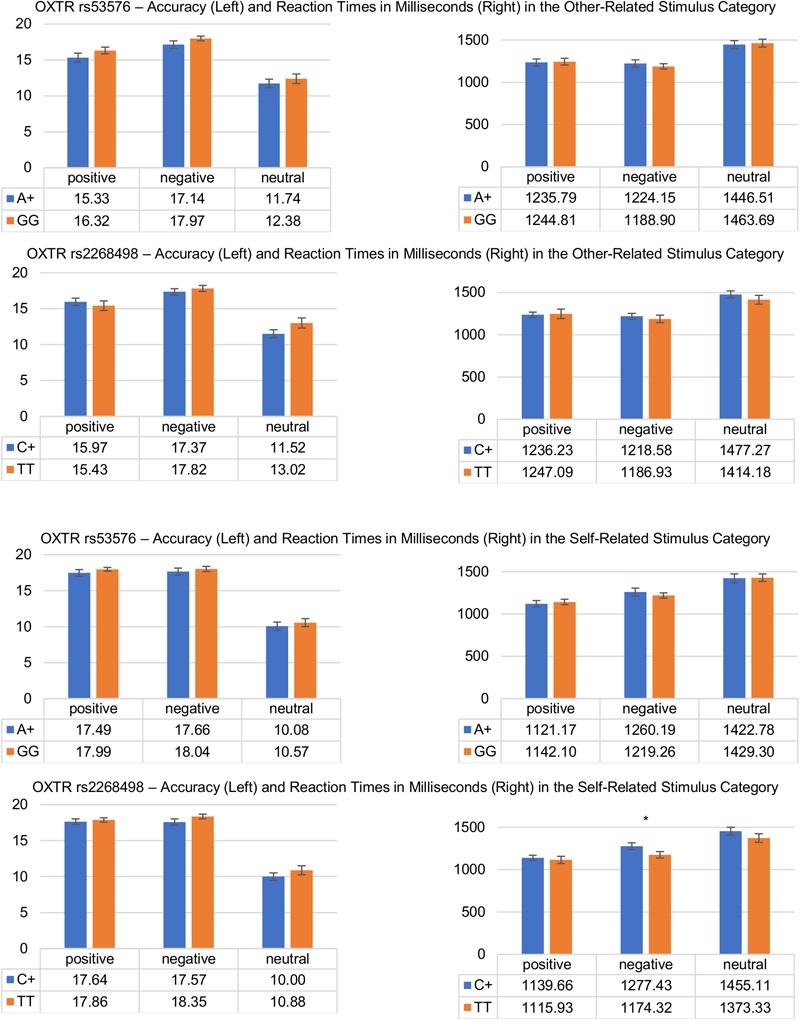
Differences in response accuracy and reaction times during the affective evaluation of other-related (upper) and self-related (lower) stimuli as a function of OXTR genotype (rs53576 and rs2268498). Vertical bars denote ±SE (^∗^*p* ≤ 0.05).

The results of the *post hoc* tests for the two SNPs are summarized in Figure 2.

### OXTR Genotypes (rs53576 and rs2268498) and Interpretation Bias

On average, all participants answered *M* = 12.17 (*SD* = 4.20) times with a “neutral” button press in the neutral stimulus categories, which is significantly less than valence-congruent answers in positive [*M* = 16.76, *SD* = 3.51; *t*(148) = 10.08; *p* ≤ 0.001] or negative [*M* = 17.66, *SD* = 3.57; *t*(48) = 13.11; *p* ≤ 0.001] stimulus categories. Moreover, neutral words were evaluated on average more often as positive than as negative [positive: *M* = 6.56, *SD* = 3.72; negative: *M* = 0.95, *SD* = 1.37; *t*(148) = 18.02; *p* ≤ 0.001], suggesting an overall tendency or bias to interpret neutral words in a positive manner (positive interpretation bias).

An independent samples *t*-test indicated that the *total interpretation bias* (positive and negative evaluations of neutral words) did not differ between A+ and GG carriers [A+ (*M* = 7.61, *SD* = 4.17) vs. GG (*M* = 7.83, *SD* = 4.09); *t*(147) = 0.007; *p* = 0.932]. Also, *the total interpretation bias* did not differ between C+ and TT carriers [C+ (*M* = 7.84, *SD* = 4.21) vs. TT (*M* = 6.86, *SD* = 3.91); *t*(147) = 1.38; *p* = 0.170]. 3 (*stimulus reference*) × 2 (*genotype A+/GG or C+/TT*) repeated measures ANOVAs were carried out to test for interactional and main effects of genotype and stimulus reference on the *interpretation bias*, revealing for the rs53576 *genotype* significant main effects of *stimulus reference* [*F*(1.65,243) = 24.95, *p* ≤ 0.001, $\eta_{p}^{2}$ = 0.145] and *genotype* [*F*(1,147) = 4.89, *p* ≤ 0.05, $\eta_{p}^{2}$ = 0.032], but no interaction between the two factors [*F*(1.65,243) = 0.586, *p* = 0.526, $\eta_{p}^{2}$ = 0.004]. For the rs2268498 *genotype* (C+ vs. TT) the repeated measures ANOVA revealed significant main effects of *stimulus reference* [*F*(1.66,244) = 19.74, *p* ≤ 0.001, $\eta_{p}^{2}$ = 0.118] and *genotype* [*F*(1,147) = 5.66, *p* ≤ 0.05, $\eta_{p}^{2}$ = 0.037], and no interaction between the two factors [*F*(1.66,244) = 1.47, *p* = 0.234, $\eta_{p}^{2}$ = 0.010].

**FIGURE 3:**
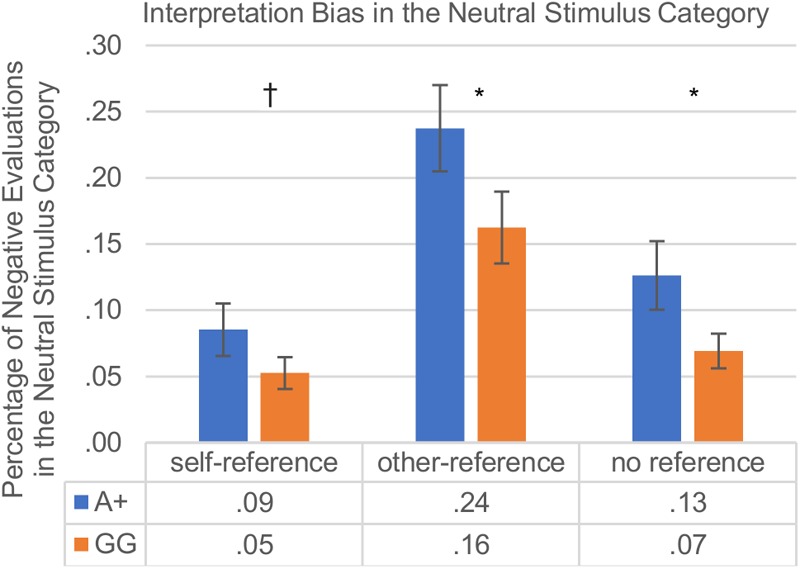
Interpretation bias in neutral words with self-reference vs. other-reference vs. no reference in percent; group comparison: rs53576-A+ vs. rs53576-GG carriers. Vertical bars denote ±SE (^†^*p* ≤ 0.1; ^∗^*p* ≤ 0.05). As described in detail under the section “Procedure” a high score (>0.5) indicates a negativity bias in the reaction towards neutral words, whereas a low score (<0.5) indicates a positivity bias.

The main effects of *genotype* were examined further in each of the reference categories separately for the two SNPs. As hypothesized, the *interpretation bias* varied with self-other reference. Regarding the rs53576, A+ carriers displayed a weaker positive interpretation bias compared to GG carriers in the other- and unreferenced stimulus categories, and also showed a tendentially weaker positive interpretation bias in the self-referenced stimulus category which did not reach significance [self-reference: A+ (*M* = 0.086, *SD* = 0.177) vs. GG (*M* = 0.053, *SD* = 0.100); *t*(128) = 1.42; *p* = 0.079; other-reference: A+ (*M* = 0.238, *SD* = 0.292) vs. GG (*M* = 0.163, *SD* = 0.226); *t*(145) = 1.77; *p* = 0.040; no reference: A+ (*M* = 0.126, *SD* = 0.231) vs. GG (*M* = 0.069, *SD* = 0.109); *t*(116) = 1.97; *p* = 0.026]. Regarding the rs2268498, participants with the C+ variants differed from TT carriers in the *interpretation bias* in the self- and in the other-referenced stimulus categories, showing a weaker positive interpretation bias in the self-reference condition and in the other-reference condition than TT carriers [self-reference: C+ (*M* = 0.084, *SD* = 0.170) vs. TT (*M* = 0.043, *SD* = 0.081); *t*(146) = 2.00; *p* = 0.047; other-reference: C+ (*M* = 0.238, *SD* = 0.290) vs. TT (*M* = 0.135, *SD* = 0.194); *t*(138) = 2.57; *p* = 0.011; no reference: C+ (*M* = 0.114, *SD* = 0.199) vs. TT (*M* = 0.072, *SD* = 0.159); *t*(147) = 1.41; *p* = 0.160]. The results of the interpretation bias are depicted in Figure 3.

### OXTR Genotype and Self-Report Measures

A+ carriers differed significantly in their scores on the personal distress subscale of the empathy scale from GG carriers [A+ (*M* = 11.04, *SD* = 3.26) vs. GG (*M* = 10.00, *SD* = 2.53); *t*(145) = 2.18; *p* = 0.031]. Besides that, all other group comparisons between A+ and GG carriers, regarding overall empathy (SPF), its corresponding subscales except “personal distress,” overall alexithymia (TAS) and its subscales, self-concept and its facets, as well as anxiety (STAI state/trait) and depression scores, were not significant (all *p* > 0.1). Regarding the SNP rs2268498, C+ and TT carriers did not differ in any of the self-report measures (all *p* > 0.1). For a detailed overview of scores and corresponding *t*-tests reported in this section, please see also Table 2.

## Discussion

This study investigated the relationship between individual differences in OXTR (rs53576 and rs2268498) genotypes and affective evaluation of emotional and neutral words with self-, other- or no personal reference. According to previous genetic studies, it is largely unknown whether genetic variations of the OXTR gene and its previously reported differences in socio-emotional information processing also hold for biologically less determined stimuli such as words, and if so, for which contents and stimulus categories affective evaluation may differ by interindividual differences in OXTR genotypes (A+ vs. GG carriers and C+ vs. TT carriers). Using a novel, yet established experimental task (the so called His-Mine paradigm), we determined whether response accuracy and reaction times as well as valence-incongruent responses to neutral words (interpretation bias) differ as a function of participants’ OXTR genotypes.

Regarding affective evaluations of positive and negative words varying in self-other reference, there was no evidence that would support an influence of participants’ OXTR genotype status on affective stimulus evaluation. Participants’ affective evaluations seemed to be unaffected by the oxytocin genotype status as was evident in the lack of any interaction between the factors *genotype* (rs53576 – A+ vs. GG or rs2268498 – C+ vs. TT) and *stimulus valence* or *stimulus reference*, regarding response accuracy as well as reaction times. Also, response accuracy and reaction times to other-related words (positive, negative or neutral) did not differ between A+ and GG or between C+ and TT carriers. This is contrary to our first hypothesis which - built on results from previous studies on non-verbal socio-emotional information processing - would predict differences in A+ and GG carriers and in C+ and TT carriers in the processing of signals relevant for interpersonal communication such as emotional information belonging to oneself or to others. One reason for the discrepancy between previous and the current findings could be that for words, the content of positive and negative self- and other-referenced stimuli is semantically obvious, easy to categorize and thus not vulnerable to misinterpretation and not dependent on individual differences in participants’ trust, emotional recognition or empathy skills that all have been shown to vary with OXTR genotype. In the present study, A+ and GG or C+ and TT carriers did not differ significantly in self-reported emotion recognition (alexithymia), self-reported trait and state anxiety or depression. Regarding empathy, significant differences could be found regarding personal emotional distress suggesting A+ carriers to be more susceptible to emotional distress than GG carriers.

According to the Component Process Model of Emotions (CPM; Ellsworth and Scherer, 2003; Sander et al., 2005) a sequential process is applied after confrontation with a stimulus. With the first step being sensory perception, appraisal afterward comprises a novelty check, a self-relevance check containing an intrinsic pleasantness check, a goal/need significance check, a coping potential check and a norm-self-compatibility check (Ellsworth and Scherer, 2003; Sander et al., 2005). Misjudgments of personally relevant information may happen at any of these stages, possibly leading to negative reactions in oneself and in others. It is reasonable to assume that the less information is provided, the more interpretative liberty is given to the individual, making neutral stimuli an empty canvas for a person’s affective evaluation, especially if presented without any obvious valence or personal reference. This is also evident in the present study: neutral words have been less often correctly evaluated as “neutral,” but instead have been interpreted as positive or negative, suggesting a genotype-independent and overall tendency to evaluate neutral stimuli in an affective fashion. Additionally, our data indicates slower reaction times for the “neutral” stimulus category, which is probably due to the (mis-) interpretation of the stimulus taking more time compared to responding in a valence-congruent fashion.

There were no differences in A+ and GG carriers or in C+ and TT carriers in overall response accuracy of the evaluation of neutral words. However, there was a general tendency to evaluate neutral stimuli more often as positive as negative (interpretation bias), which was modulated by participants’ OXTR genotype. More specifically, rs53576 A+ carriers showed a weaker positive interpretation bias compared to GG carriers during affective evaluation of other- and unreferenced neutral words. Rs2268498 C+ carriers showed a weaker positive interpretation bias during affective evaluation of self- and other-referenced neutral words compared to TT carriers. One limitation of the present finding regarding the interpretation bias could be the overall small trial number included for its calculation. Future studies might therefore include overall larger trial numbers per word category or selectively incorporate more neutral trials per word category to replicate and validate the present observation of an interpretation bias and its modulation by the OXTR genotypes.

One possible explanation of these findings could be that neutral words constitute the category with the least information, making their affective evaluation more susceptible to misinterpretation and therefore more ambiguous than affective evaluation of emotional words. Given that both SNPs did affect valence judgments of neutral words in a slightly different way, this supports previous observations (see the section “Introduction” for details) that variance in social behavior captured by the two different SNPs – OXTR rs53576 and rs2268498 – is not mutually interchangeable. In addition, the results support a role of the two SNPs in the appraisal of neutral content, with A+ carriers being less prone to positive interpretation biases for other- and unreferenced neutral words than GG carriers (rs53576) and C+ carriers being less prone or sensitive to positive interpretation biases for self- and other-related neutral words than TT carriers (rs2268498).

At the moment, we can only speculate about the adaptive value of these OXTR genotype-related differences in the interpretation bias: from an evolutionary perspective, both, a reduced as well as an increased positive interpretation bias for unreferenced as well as for self-related or other-related content might have adaptive value in terms of well-being, whereas a negative interpretation bias may be associated with negative effects on well-being and affective disorders as has been shown for depression and anxiety (e.g., Huppert et al., 2003; Amir et al., 2005; Wisco and Nolen-Hoeksema, 2010; Berna et al., 2011). Given that especially A+ genotypes have been reported to be more prone to depressive symptoms (Saphire-Bernstein et al., 2011) and since in the present study only healthy participants were included, it would be interesting to investigate the interpretation bias in depression- or anxiety-prone subjects with varying OXTR genotype status in future studies. On a related note, additionally including participants with an ASD diagnosis in the present experimental design may shed additional light onto the relationship of the OXTR gene and social behavior, as differences in OXTR genotypes and ASD have been reported for the SNP rs2268498 (Montag et al., 2017), and for rs53576 (in a Chinese population; Wu et al., 2005), which, however, seem to be dependent on factors such as ethnicity (Jacob et al., 2007), or OXTR DNA methylation (Rijlaarsdam et al., 2017). It could also be interesting to extend the present paradigm to (semi-)realistic interactional scenarios to fully understand extent and meaning of the reported interpretation bias.

Regarding the selection of the SNPs (rs53576 and rs2268498) of the OXTR gene, the two SNPs of the present study were chosen according to the previous literature. So far, relatively little is known about the impact of both OXTR SNPs on the biochemical pathways underlying the oxytocinergic neurotransmission. Regarding rs2268498 (C+ vs. TT), its direct relation with oxytocin expression has been recently demonstrated in a functional neuroimaging study in humans (Reuter et al., 2017). Possibly, such molecular mechanisms may play a role in the context of our observed association between OXTR rs2268498 and the interpretation bias, whereas direct links between behavior, oxytocin release or oxytocin expression and OXTR rs53576 genotype have still not be proven in humans. Nevertheless, recent studies suggest that the OXTR rs53576 genotype – although being located in an intronic region of the OXTR gene – might still have an impact on gene expression: in prairie voles it has been demonstrated that genetic variation near *cis*/regulatory elements of the OXTR gene could contribute to individual differences in gene expression in brain areas of relevance for social attachment such as the *nucleus accumbens* (NAcc) (King et al., 2016). Furthermore, individual differences in hypothalamic-limbic areas of the brain depending on genetic information of rs53576 has been shown by Tost et al. (2010) providing further evidence, that this SNP somehow is linked to brain processes, perhaps indirectly via linkage to other genetic variants.

## Conclusion

As we outlined in the “Introduction” and in the “Discussion” sections above, the literature points towards functional connections between neuronal areas associated with oxytocin production and the limbic system, including the amygdala and the hippocampus (Landgraf and Neumann, 2004; MacDonald and MacDonald, 2010). Previous studies have also shown an involvement of the amygdala, an area rich in OXTRs, in the processing of affective language (e.g., Kuchinke et al., 2005; Herbert et al., 2009), in the processing of emotional words related to self and others (Herbert et al., 2011b). Moreover, previous studies suggested a relationship between OXTR genotypes and the aforementioned brain structures (e.g., Tost et al., 2010). Therefore, to further evaluate the mechanisms of social and affective information processing, it would be interesting to replicate the findings of this study utilizing neuroimaging methods such as fMRI to gain further insight into possibly genotype-dependent neural activity in the processing of self- and other-related or personally unrelated emotional words particularly also regarding the choices of valence-incongruent responses (interpretation bias). In general, effects of OXTR genotypes on behavior are small and therefore larger sample sizes than the one available in the present study are needed to robustly track and replicate the present observations regarding genetic effects.

While delivering first insight into the association between different OXTR genotypes (rs53576 and rs2268498) and affective processing of verbal stimuli, the presented study also has its limitations: First, real-world interactions and interpersonal communication are often much more complex than the evaluation of basal, verbal stimuli, as employed in this study. Therefore, although the present paradigm and study investigated important parts of the appraisal processes in people with different OXTR genetic status, future studies will need to take the full process and sequences of stimulus appraisal as for instance described in the Component Process Model of Emotions (CPM; Ellsworth and Scherer, 2003; Sander et al., 2005) into consideration. Additionally, although the previous literature describes A+ (rs53576) or C+ (rs2268498) carriers as “deficient” in the processing of socio-emotional information, one should consider that this might simply be conventional nomenclature and that this polymorphism probably has adaptive value to it as well (i.e., what might be called deficits in some areas might be an advantage in other aspects of life). The present findings revealing individual differences in the interpretation bias between A+ and GG carriers and between C+ and TT carriers in a healthy participant sample might speak in favor of this assumption.

## Data Availability Statement

The data supporting the conclusions of this manuscript will be made available by the authors, without undue reservation, to any qualified researcher. Due to the informed consent form in which the possibility of raw data being published online was not explicitly stated, only group-level data, as it is provided in this manuscript, will be made accessible.

## Author Contributions

FM prepared the manuscript and CH revised it for content. FM recorded the data and preprocessed and analyzed the results under the supervision of CH. FM prepared the tables and figures. CH programmed and designed the paradigm. CM and his team were responsible for the genetic analysis (preprocessing). All authors approved the final version of the manuscript.

## Conflict of Interest Statement

The authors declare that the research was conducted in the absence of any commercial or financial relationships that could be construed as a potential conflict of interest.

## Footnotes

1 https://www.uni-ulm.de/einrichtungen/ethikkommission-der-universitaet-ulm/

2 Regarding handedness, exclusion of left-handers might not have been necessary because participants were instructed to reply with their dominant hand and therefore, left- or right-handedness seem not to bias the behavioral results in the His-Mine Paradigm (e.g., Meixner and Herbert, 2018). However, since an association between oxytocin and testosterone levels on the one hand and testosterone in pregnancy and handedness on the other hand cannot be ignored and has occasionally been reported in the literature, left-handers were excluded from the analysis to guarantee a homogenous sample.

3 We employed only the male pronoun “his” (German: sein/seine). In the German language, “her” (ihr/ihre) is almost indiscernible from “their” (Ihr/Ihre) and was therefore not included due its possible ambiguity.

4 The full list of stimuli is available from the corresponding author upon request.

5 http://www.neurobs.com

6 For reasons of brevity, the term “(response) accuracy” will be used. The term “response accuracy” thus refers to the number of valence-congruent responses.
